# Consensus Paper. Cerebellar Reserve: From Cerebellar Physiology to Cerebellar Disorders

**DOI:** 10.1007/s12311-019-01091-9

**Published:** 2019-12-26

**Authors:** H. Mitoma, A. Buffo, F. Gelfo, X. Guell, E. Fucà, S. Kakei, J. Lee, M. Manto, L. Petrosini, A.G. Shaikh, J.D. Schmahmann

**Affiliations:** 1 Medical Education Promotion Center, Tokyo Medical University, Tokyo, Japan; 2 Department of Neuroscience Rita Levi-Montalcini, University of Turin, 10126 Turin, Italy; 3 Neuroscience Institute Cavalieri Ottolenghi, 10043 Orbassano, Italy; 4 Department of Human Sciences, Guglielmo Marconi University, 00193 Rome, Italy; 5 IRCCS Fondazione Santa Lucia, 00179 Rome, Italy; 6 Department of Neurology, Massachusetts General Hospital, Ataxia Unit, Cognitive Behavioral Neurology Unit, Laboratory for Neuroanatomy and Cerebellar Neurobiology, Harvard Medical School, Boston, USA; 7 McGovern Institute for Brain Research, Massachusetts Institute of Technology, Cambridge, USA; 8 Child and Adolescent Neuropsychiatry Unit, Bambino Gesù Children’s Hospital, 00165 Rome, Italy; 9 Tokyo Metropolitan Institute of Medical Science, Tokyo, Japan; 10 Komatsu University, Komatsu, Japan; 11 Unité des Ataxies Cérébelleuses, Service de Neurologie, CHU-Charleroi, 6000 Charleroi, Belgium; 12 Service des Neurosciences, University of Mons, 7000 Mons, Belgium; 13 Louis Stokes Cleveland VA Medical Center, University Hospitals Cleveland Medical Center, Cleveland, OH USA

**Keywords:** Cerebellum, Cerebellar reserve, Cerebellar ataxias, Eye movement, Saccade, Predictive control, Cerebellar cognitive affective syndrome, Autophagy, Environmental enrichment, Dendritic spines, Neuromodulation therapy

## Abstract

Cerebellar reserve refers to the capacity of the cerebellum to compensate for tissue damage or loss of function resulting from many different etiologies. When the inciting event produces acute focal damage (e.g., stroke, trauma), impaired cerebellar function may be compensated for by other cerebellar areas or by extracerebellar structures (i.e., structural cerebellar reserve). In contrast, when pathological changes compromise cerebellar neuronal integrity gradually leading to cell death (e.g., metabolic and immune-mediated cerebellar ataxias, neurodegenerative ataxias), it is possible that the affected area itself can compensate for the slowly evolving cerebellar lesion (i.e., functional cerebellar reserve). Here, we examine cerebellar reserve from the perspective of the three cornerstones of clinical ataxiology: control of ocular movements, coordination of voluntary axial and appendicular movements, and cognitive functions. Current evidence indicates that cerebellar reserve is potentiated by environmental enrichment through the mechanisms of autophagy and synaptogenesis, suggesting that cerebellar reserve is not rigid or fixed, but exhibits plasticity potentiated by experience. These conclusions have therapeutic implications. During the period when cerebellar reserve is preserved, treatments should be directed at stopping disease progression and/or limiting the pathological process. Simultaneously, cerebellar reserve may be potentiated using multiple approaches. Potentiation of cerebellar reserve may lead to compensation and restoration of function in the setting of cerebellar diseases, and also in disorders primarily of the cerebral hemispheres by enhancing cerebellar mechanisms of action. It therefore appears that cerebellar reserve, and the underlying plasticity of cerebellar microcircuitry that enables it, may be of critical neurobiological importance to a wide range of neurological/neuropsychiatric conditions.

## Introduction

### Definition of Cerebellar Reserve

Self-repair is an outstanding feature of the cerebellar system that enables responses to lesions such as stroke, surgical ablation, neoplasms, and neurodegeneration [1–3]. This unique property was first described in animals by Luigi Luciani (1891) [3] and in patients with gunshot injuries by Gordon Holmes (1917) [4]. Holmes described the recovery process of motor incoordination in two patients; one had a limited lesion in the cerebellar lateral lobe, and the other had a larger and mesial lesion in the same area. Despite considerable damage, both patients walked with stability after 58 days and 71 days respectively. Interestingly, there was no difference between their gait, despite the marked difference in the extent of the two lesions.

Here, we define cerebellar reserve as the capacity of the cerebellum to compensate and restore function in response to pathology. This property can result in clinical tolerance to pathology and reversibility after its removal. The notion of reserve was initially proposed to account for differences in susceptibility of the cerebral cortex to aging or pathological damage [5–9]. In this regard, reserve is defined as a moderator between pathology and outcome [5]. Stern (2012) defined three types of reserve, brain (i.e., anatomical) reserve, cognitive (i.e., functional) reserve, and neural (i.e., network-based) reserve [5–7]. Since brain reserve correlates with the number of remaining intact/undamaged neurons and synapses after damage/pathology, it is morphological and quantitative in nature. In contrast, cognitive reserve relates to functional activity, such as utilizing “pre-existing cognitive approaches” or “enlisting compensatory approaches”[5]. Since these functional activities depend on the integrity of brain networks, it has been proposed that neural reserve is a consequence of “the efficacy of neural circuitry and the ability to process information” [8], and that these mechanisms contribute to compensation for aging or dementia-related processes [5–9]. In our consideration of cerebellar reserve, we draw attention to this classical meaning of resilience to impairment, and we also highlight the notion of reversibility [10] (Table 1). Reversibility may be enabled by the unique morphological and functional features of the cerebellum with its stereotyped and highly geometric, lattice-like architecture, and its enormous number of neurons; between 60 and 70% of the brain’s neurons are in the cerebellum (about 25 million Purkinje cells and as many as 70 billion granule cells) [11, 12].

**Table 1:** Notions of reserve

**Classical meaning of “Reserve”**	
The notion of reserve was introduced in the sense of resistance to aging or dementia. [5–9]	
***Brain reserve***	
The number of remaining intact/undamaged neurons and synapses, thus the term has morphological and quantitative meaning.	
***Functional reserve***	
The utilization of pre-existing cognitive approaches, thus the term has functional meaning.	
***Neural reserve***	
The efficacy of neural circuitry and the ability to process information, thus the term focuses on brain network functioning.	
**Definition of “Cerebellar reserve”**	
Cerebellar reserve refers to the classical meaning of resilience to impairment and also to the capacity for reversibility. It is defined as the capacity of the cerebellum to compensate and restore function in response to pathology.	
Cerebellar reserve is conceptualized as the result of two complimentary mechanisms, depending on the underlying etiology	
***Structural cerebellar reserve***	
In cases when etiologies elicit acute structural damage and cell death in a focal area (e.g., stroke and injuries).	
Compensation by the remaining intact cerebellar areas outside the lesion	
***Functional cerebellar reserve***	
In cases when the neuropathology produces cerebellar neuronal dysfunction gradually leading to cell death (e.g., immune-mediated cerebellar ataxias, metabolic ataxias and neurodegenerative ataxias).	
Functional restoration and compensation within the lesion	

### Two Aspects of Cerebellar Reserve Depending on Etiologies

Cerebellar restoration and compensation following lesions may be conceptualized as the result of two complementary mechanisms, depending on the underlying etiology. Following acute, focal structural damage (e.g., stroke, trauma), impaired cerebellar function(s) may be compensated for by other cerebellar or extracerebellar areas not directly affected by the lesion, that is, structural cerebellar reserve. In contrast, when the neuropathology affects cerebellar neurons leading eventually to cell death, the affected area itself may contribute to the preservation of cerebellar function. This may occur in the setting of immune-mediated cerebellar ataxias (IMCAs), metabolic ataxias, and neurodegenerative ataxias, all of which are progressive and generally diffuse throughout the cerebellum. Cerebellar restoration and compensation may occur through functional reorganization, such as intracellular defensive molecular mechanisms to avoid cell death, and facilitatory plasticity at weakened synaptic transmissions. This type of restoration and compensation is possible only when the capacity for functional reorganization is preserved, hence, in the presence of functional cerebellar reserve.

### Clinical and Pathophysiological Characteristics of Cerebellar Reserve

Here, we first address the pathophysiological features underlying structural and functional cerebellar reserve. We discuss the changes that develop following experimentally induced cerebellar lesions, including the recovery processes which represent the basis for the proposed notion of structural cerebellar reserve in the clinical domain. The results of these animal experiments suggest that the neural compensatory response includes the process of rebuilding neural circuits for substitution or relearning. Clinical observations in immune-mediated cerebellar ataxias provide insights into common pathophysiological processes underlying functional cerebellar reserve. Second, we examine the processes of restoration and compensation following different types of lesions, and address the clinical quantification of cerebellar reserve. In this manner, we explore cerebellar reserve for each of the three cornerstones of clinical ataxiology: control of ocular movements, coordination of voluntary axial and appendicular movements, and cognitive functions. Finally, we review the evidence that cerebellar reserve is potentiated by complex environmental stimulation as occurs in enriched environments, through mechanisms of autophagy and synaptogenesis. These experimental observations lead to the conclusion that cerebellar reserve is not rigid or fixed, but rather exhibits plasticity that can be potentiated by experience.

## Structural Cerebellar Reserve: Implications From Lesion Studies in Animals. Compensation, Recovery, and Cerebellar Reserve (Manto M)

Experimental studies, performed mainly in rodents and monkeys, have demonstrated that cerebellar lesions are followed by a substantial recovery, even when the lesions are extensive. This partial or apparently complete recovery is associated with a reorganization of cerebellar and extracerebellar networks. The timing of cerebellar lesions through lifespan is a key-factor. Lesions occurring during development do not follow a similar recovery course than lesions performed at the adult stage [13–17]. The general rule is that lesions performed in the perinatal period will evolve and change with age with a suggestive delayed handicap period, whereas their immediate consequences can be predicted in adults according to the size and site of the lesion [14]. Motor deficits following cerebellar lesions (before 10th day in rats) become visible after the 15th day as a consequence of the immaturity of the cerebellum [14]. This is explained by a circuitry which is not fully established at the early period. In addition, at an early stage, another important feature is the common lack of correlation between the severity of deficits and the size of the lesion: a large lesion may induce only subtle deficits. Therefore, the characterization of cerebellar reserve in young animals is not an easy task and it is necessary to consider the timing of occurrence of the damage from the developmental period to adulthood. Furthermore, at a time where all the community has accepted the critical roles of the cerebellum in cognitive operations [18], there is a consensus that cognitive cerebellar reserve is hard to quantify in animals for obvious reasons, even if tools have been developed to estimate the recovery or secondary decompensation [19]. The rescue from an initial injury is also dependent on environmental enrichment, a factor complicating the quantification of the reserve [20].

### Focal Administration of Toxics

Cerebellar lesions induced by administration of toxic agents locally in the cerebellar circuitry induce deficits of voluntary movements in monkeys [21]. In particular, kainic acid injected in the cerebellar nuclei (interpositus/dentate) causes deficits of proximal and distal voluntary movements, which recover over time. This recovery (compensation) is followed by a decompensation when the sensory cortex is removed secondarily [21]. Removal of the sensory cortex before the cerebellar lesion increases the cerebellar deficits and severely reduces the recovery, indicating a strong functional link between cerebellum and sensory cortex.

### The model of Hemicerebellectomy and Total Cerebellectomy

In rats, both the hemicerebellectomy (HCb) and the full cerebellectomy models are followed by a recovery of deficits after a few weeks up to a few months. Limb hyperflexion, wide-based locomotion, and the tendency to side falls are common after cerebellectomy, whereas tremor and body tilt develop after HCb [22]. Some studies have shown a partial recovery 6 months after the lesion [23].

Glutamatergic transmission is facilitated in the contralateral striatum following HCb in rats. Pharmacological blockade of *N*-methyl-d-aspartate (NMDA) receptors with MK-801 impairs the rearrangement of excitatory synapses in the striatum and impacts on the compensation from motor disturbances [24]. These observations underline the importance of neurotransmitters in the process of compensation.

Conversely, cerebellum is a key player for the recovery after cerebral hemispherectomy [25]. The sequence of cerebral hemispherectomy and HCb impacts the recovery process: if the HCb is performed before the cerebral hemispherectomy, the compensation is lower as compared to the reverse sequence.

Ipsilateral aberrant cerebello-rubral projections develop following neonatal HCb [26]. This novel pathway mirrors the topographic arrangement of the normal ipsilateral input at a synaptic level. This aberrant rewiring is explained by a response to deafferentation of red nucleus that is not specific to genuine cerebellar lesions. It is interesting to note that spinovestibular pathways also show a remodeling following HCb in newborn rats [27]. The fibers are redirected toward vestibular nuclei instead of the cerebellum.

Neonatal ablation of the cerebellar cortex in monkey does not cause oculomotor deficits at the adult stage, provided the cerebellar nuclei are intact [28]. When cerebellar nuclei are removed, residual deficits will persist. The preservation of nucleofugal pathways is required for an apparent full compensation.

### The compensation Following Transection of Cerebellar Peduncles

Neonatal transection of cerebellar peduncles is rapidly followed by a reinnervation of the deprived cerebellar cortex by olivocerebellar projections with reformation of a sagittal striped pattern [29]. A specific reinnervation occurs also in cerebellar nuclei, with the aim of rebuilding a correct map [30]. The climbing fiber projections from the inferior olive are critical to gate sensory inputs with temporal precision [31]. Both excitatory and inhibitory transmitters in vestibular nuclei show profound changes after peduncle transection [32].

In conclusion, the neurobiological substrates of cerebellar compensation include a non-random remodeling of both cerebellar networks and cerebello-striato-cerebral networks but also changes in spinovestibular projections and cerebello-rubral projections with structural plasticity. Experimental studies highlight the powerful compensatory properties of the brain following a cerebellar lesion. Thanks to a large repertoire of plastic mechanisms within the cerebellar circuitry itself especially at the cerebellar cortical level, compensation also involves purely local responses in the cerebellar cortex and in cerebellar nuclei [33]. Cerebellum is also recruited in the mechanism of compensation after an extracerebellar injury, including for lesions of the peripheral nervous system such as transection of nerves [14]. Cerebellar input is mandatory for relearning of motor functions after hemispherectomy removing the cerebral sensorimotor cortex [34]. The links between learning, compensation/decompensation, and cerebellar reserve require specific studies. This is a critical point at a time when techniques such as transcranial direct current stimulation (tDCS) or repetitive transcranial magnetic stimulation (rTMS) are increasingly used to promote recovery [35]. One example of the importance of the delineation of cerebellar reserve comes from the observation that animals undergoing both cortical hemispherectomy and contralateral cerebellar resection cannot relearn motor tasks [34]. This clarification will impact greatly on rehabilitation techniques in human cerebellar and extracerebellar disorders. The concepts of (1) substitutional neuronal circuits, (2) relearning via normally unused circuits, and (3) rebuilding of neuronal circuits by sprouting and regeneration were proposed by Sasaki and Gemba three decades ago [36]. There is still a major need to clarify how they relate to the cerebellar reserve.

## Functional Cerebellar Reserve: Implications From Immune-Mediated Cerebellar Ataxias (Mitoma H)

After damage induced by vascular disease, trauma, or local tumors, clinicians sometimes witness surprising improvement in limb motor incoordination and unstable gait. The recovery process after motor incoordination is presumed to involve deficit compensation by the residual damage-free area of the cerebellum, as discussed in the previous section (structural cerebellar reserve) [37]. For example, motor rehabilitation facilitates these compensatory processes [37].

Importantly, recovery of motor symptoms in cerebellar ataxias (CAs) has been noticed even in patients with diffuse and progressive cerebellar damage, such as IMCAs, metabolic ataxias, and degenerative CAs [10]. Furthermore, adequate immunotherapy improves motor CAs partially and even sometimes completely in some patients with IMCAs. Also, motor rehabilitation is effective in patients with degenerative CAs [37]. Thus, the neural structures involved in the restoration process are not only present in the residual damage-free cerebellum with limited and transient lesions but also in the cerebellum with diffuse and progressive lesions. These neural entities form *the functional cerebellar reserve*.

Systematic reviews of the IMCAs can provide interesting therapeutic strategy-related notions; (1) restorable stage and non-restorable stage, and (2) switching from a functional disorder to cell death [10, 38]. IMCAs include paraneoplastic cerebellar degenerations, gluten ataxia, and anti-GAD65 antibody-associated cerebellar ataxia (anti-GAD65 Ab-associated CA) [39–41].

### Restorable Stage: a Stage Characterized by Preservation of Cerebellar Reserve

**Fig. 1:**
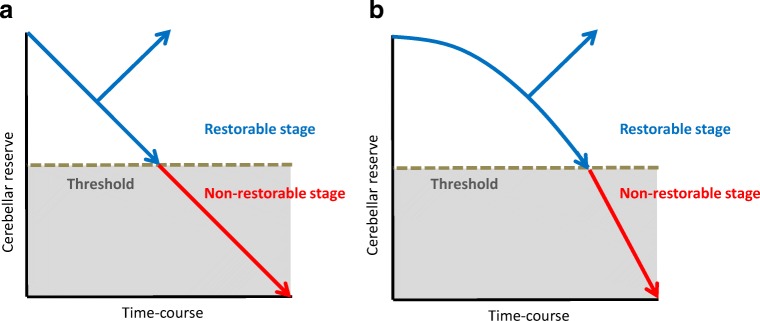
Schematic diagram explaining the concept and relationship between the restorable stage and cerebellar reserve

Two factors determine the clinical course after immunotherapy (Fig. 1a).

1. Response and no response. Immunotherapy can halt the process of immune progression in certain etiologies but not in others. For example, avoidance of gluten curtails the progression of gluten ataxia, whereas surgical excision of neoplasms and administration of a combination of immunotherapies (e.g., corticosteroids, intravenous immunoglobulins, and immunosuppressants) cannot halt the progression of paraneoplastic cerebellar degenerations. In anti-GAD65Ab-associated CA, the immune response is more pronounced in the acute onset subtype than in the chronic subtype.
2. Restorable or non-restorable stage. Induction of immunotherapy can be followed by two different clinical outcomes: patients at early stage of the disease, with normal or mildly atrophied cerebellum, show partial or full recovery. In contrast, patients with advanced stage, most of whom have evident cerebellar atrophy, show no improvement, although the CAs remain stable. These features are observed in almost all types of IMCAs, suggesting the existence of a threshold that differentiates restorable stage from non-restorable stage.

The restorable stage is defined as the period associated with intact cerebellar reversal capacity, i.e., cerebellar reserve. Accordingly, any therapeutic strategy for IMCAs should aim to stall the process of autoimmune progression.

### Switch From Functional Disorder to Cell Death

Preservation of cerebellar motor reserve is noted in IMCAs, compared with other etiologies, such as metabolic ataxias and, especially, degenerative ataxias. The reason for disease progression is either the chronic nature of IMCAs or a switch from a functional disorder to cell death.

The role of switching is illustrated in anti-GAD65 Ab-associated CA, a subtype of IMCAs. Anti-GAD65 Ab accesses GAD65 in the synaptic terminals of GABAergic interneurons, resulting in reduction of GAD65 contents in GABA-containing vesicles, which reduces GABA release. The resultant decrease in GABA release can reduce inhibitory postsynaptic currents through GABA_A_ receptors and attenuate presynaptic inhibition of glutamate release from neighboring excitatory synapses. In other words, the decrease in GABA induces an imbalance in neurotransmitters, decreasing GABA and increasing glutamate, with resultant inappropriate neurotransmitter output [38]. This pathophysiological impairment ultimately leads to excitotoxicity-induced cell death. Microglia activated by the excess glutamate increase the non-synaptic release of glutamate through the xc(-) system, and secrete TNF-α, which in turn inhibits the uptake of extracellular glutamate from excitatory amino acid transporters (EAATs) [38]. Thus, the relative increase in glutamate release is potentiated by positive feedback, and ultimately results in excitotoxicity-induced cell death. On the other hand, excessive glutamate-induced activation of NMDA receptors and the associated Ca^2+^ influx results in stimulation of calpain I and nNOS [42]. This causes DNA damage and formation of ONOO^−^ due to excess NO (nitrosative stress), respectively. The excess of Ca^2+^ also causes mitochondrial dysfunction, ultimately leading to energy crisis and ER stress [42]. Consistent with this scenario, cell loss is evident in the advanced stage [38]. This excessive glutamate-induced positive feedback progression can be summarized in the schematic diagram shown in Fig. 1b rather than that in Fig. 1a. Fig. 1b shows accelerated loss of cerebellar reserve; cerebellar reserve diminishes slowly at the initial stage, but is followed by a steep loss in the advanced stage (Fig. 1b).

In summary, the switch from functional disorder to cell death is the likely pathophysiological background underlying disease progression of IMCAs from restorable stage to non-restorable stage.

### From IMCAs to Generalization

It is uncertain whether the above changes can be generalized to other etiologies of CA. For example, in stroke, the infarct core is surrounded by the hypoxic area (ischemic penumbra) and the ischemic brain area contains functionally silent neurons that can be rescued before they undergo cell death [43]. In addition, in the early stages of degenerative diseases, functional impairment (e.g., synaptic dysfunction or signal flow in cerebellar circuits) that precedes degenerative cell loss can advance to clinically evident CAs [44, 45]. In general, the following therapeutic strategy can be applied irrespective of etiology: (1) curative treatments should be designed in the initial stages to minimize damage to the cerebellum while cerebellar impairment is still within the frame of functional disorder with adequate cerebellar reserve; (2) neuromodulation therapy should be followed to facilitate cerebellar reserve. Neuromodulation therapies include intensive motor rehabilitation [37], certain medications (aminopyridine) [46], or noninvasive cerebellar stimulation [47]. In this context, the near-future is promising with the introduction of RNA therapies and neurotransplantation [48–50].

With regard to neurotransplantation, genuine replacement is considered difficult, since cell differentiation and synaptic formation are necessary to establish functional circuitries; highly integrated reproduction of cerebellar anatomy is not a simple process [48–50]. Instead, recent studies have shown that grafted cells rescue surviving cells from neurodegeneration by exerting trophic effects, supporting mitochondrial function, modulating neuroinflammation, stimulating endogenous regenerative processes, and facilitating cerebellar compensatory properties enabled by neural plasticity [48–50]. Thus, reinforcement of cerebellar reserve and prolongation of the restorable stage can be envisioned as future endpoints of neurotransplantation.

## Cerebellar Ocular Reserve (Shaikh AG)

Cerebellar reserve can be classified into two categories. One category is functional reserve where there is no permanent structural damage to the neural substrate but the affected area operates at a suboptimal state. The second category is structural cerebellar reserve, where the affected neural substrate acquires the structural damage and the compensation for the loss happens at the residual substance in the same anatomical region or its functional counterpart elsewhere in the brain. Here, we will discuss examples for both types of cerebellar ocular reserve.

### Structural Cerebellar Ocular Motor Reserve

We utilize eye movements as a model for cerebellar motor reserve since the ocular motor physiology is well understood, the eye movements are accessible for non-invasive quantitative measurements and the focal excision models in macaques have carefully measured ocular motor function at various stages after the cerebellar lesion The excision of flocculus and paraflocculus resulted in an animal model of classic floccular syndrome where the animal had downbeat nystagmus, gaze-evoked nystagmus, and impairment in the cancellation of the vestibulo-ocular reflex (VOR) [51]. Although the primary goal of this classic study was to understand the role of flocculus in the physiology of ocular motor and vestibular function, here we extrapolate their findings to understand how the focal lesion affecting the flocculus and paraflocculus were compensated over time. In order to describe the ocular motor cerebellar reserve after focal flocculus/paraflocculus lesion, we will classify the eye movement behavior into four broad categories—rapid eye movements that shift gaze from one target to the other (saccades), gaze-holding, smooth tracking of the moving target (pursuit), and VOR. Immediately after flocculectomy, there was a robust change in the ocular pursuit system. The pursuit eye movements were not as fast as the target to be followed, resulting in reduction of the pursuit gain (velocity of the eye/velocity of the target). Such reduction in pursuit gain caused frequent retinal error as the target of interest moved away from the fovea; as a result, the eyes triggered saccades which corrected misplaced foveal location of the target. The fidelity of the pursuit system improved over time after flocculectomy. In the immediate post-lesion phase, the pursuit gain increased over a period of several months. These results suggest that flocculus is critical for ocular pursuit; however, when there is a structural damage to flocculus, other areas of the cerebellum provide adequate compensation for the lost floccular function.

Gaze holding is another system that was studied. In order to appropriately capture the visual signal, the image of interest should remain stable on the fovea. In eccentric gaze orientation, there is excessive elastic dragging force from the orbital connective tissue causing drift toward the center, which is counteracted actively by the neural integrators that convert pulses of neural discharge (i.e., the eye velocity signal) to steady-state neural firing (i.e., the eye position signal) under cerebellar supervision. Experimental flocculus lesions are thought to result in suboptimal neural integration and consequent centrally directed drifts during eccentric position the eye—i.e., the gaze-evoked nystagmus [51]. The time constant of exponentially drifting eye position characterizing gaze-evoked nystagmus is the typical measure of gaze-holding function, and is the basis of the quantitative test measuring the fidelity of neural integrator and in particular the influence of cerebellum on such physiology. Immediately after the flocculus lesion, the time constant was reduced and subsequently there were robust drifts of gaze-evoked nystagmus [51]. However, there was an increase in time constant several months after the lesion suggesting recovered function of the neural integrator. One of the alternate ways the brain may compensate for the gaze-evoked nystagmus is by generating bias [51]. While counteracting the slow-phase velocity of the gaze-evoked nystagmus in eccentric (non-null) orientations, when the eyes are in the null orientation, the bias leads to nystagmus with a slow-phase in the direction opposite to that of the preceding segment of gaze-evoked nystagmus. This phenomenon, called rebound nystagmus, is therefore frequently seen in the straight-ahead orientation (most typical null orientation) after the eyes return from the eccentric position. Rebound nystagmus is a transient phenomenon, it dampens after a short duration.

The third class of eye movements, saccades, was unimpaired after flocculectomy. The fourth class of eye movements, the VOR, assures steady object position on the fovea in the presence of head movement. This reflex is typically measured by its gain, that is, the ratio of eye and head velocity. Ideal values of gain of VOR are around 1, but it was abnormally increased in immediate post-lesion phase after flocculectomy. Unlike pursuit or gaze-holding function, the VOR gain impairment remained abnormal throughout the post-lesion course. These results suggested that the flocculus is the exclusive brain region for control of VOR gain, and in its absence there is no neural substrate to offer compensation—in other words, the cerebellar reserve for VOR is limited.

The dorsal cerebellar vermis (lobules VI–VII, also known as oculomotor vermis) has long been implicated in motor control of saccades and pursuit eye movements [52–60]. The focal experimental lesions of the dorsal vermis are known to cause dysmetria of saccades and impairment in the pursuit function, indicating the role of this structure in online control of the ocular motor behavior [52, 61, 62]. The influence of the dorsal vermis on saccade is determined by its impact on the saccade accuracy, latency, velocity, and acceleration. Immediately after the structural lesion of the ocular motor vermis, the accuracy of the saccade was disrupted [52]. Accuracy, measured as gain of saccade, was reduced to about 50% of its normal value in early phase after the focal dorsal vermis lesion [52]. There was not only a reduction in the accuracy of saccade from trial to trial, but the amplitude variability also increased to twice as much in immediate post-lesion phase. The variability was higher when the hypometria was larger [52]. In late (3–4 month) post lesion phase, there was some recovery of saccade latency and variability but also enduring dysmetria. The latency of saccade measured by the time difference between the onset of target jump and initial eye movement was also affected by the dorsal vermis lesion. In initial post-lesion phase, there was a 50% increase in latency, in late phase (3–4 months after the lesion) there was some recovery in latency, but there was no complete compensation to normal. Saccade dynamics, the comparison of saccade amplitude with corresponding velocity and acceleration (i.e., the main sequence) was variably affected by the dorsal vermis lesion. The focal dorsal vermis lesion did not change the relationship in one animal, but in two of them there was a reduction in the saccade velocity. There was no change in coherence between the saccade velocity, acceleration, and amplitude [52].

The dorsal vermis lesion also had an important consequence on pursuit eye movements. Pursuits after dorsal vermis lesion were tested in two different paradigms. In one paradigm, the steady-state gain (eye velocity/target velocity) was measured in target movement that had triangular or sinusoidal trajectory. The second paradigm was step-ramp where stationary target jumps backwards (step) and then moves at slow velocity (ramp). The unique aspect of step-ramp was that during first 100 ms of the ramp the motor system does not utilize visual (closed-loop) information. Hence, the first 100 ms of pursuit eye movement during step ramp condition is a “pure” test of ocular motor behavior during pursuit. Focal lesion of the dorsal vermis affected both paradigms, the open and closed-loop of the pursuit system. Steady-state gain tracking the target in triangular shape showed robust reduction of pursuit gain in the post lesion phase. Over 3–4 months post-lesion, there was a compensation and an increase in pursuit gain, but reversal to normal only happened for slower velocity tracking. The extent of lesion also determined the reversibility (the amount of compensation) of the pursuit gain. It is believed that the closed loop pursuit gain is the function of the dorsal vermis and the areas around it; as a consequence, the larger excision of the neural substance from the dorsal vermis leads to irreversible loss of function. In contrast, immediately after the dorsal vermis lesion, there was a reduction in the open loop acceleration; the reduction was reversible but again the extent of reversibility was determined by the size of the lesion. Hence, it is predicted that cerebellar reserve for the open loop phase is limited to the dorsal vermis or at the most to the area in proximity to the dorsal vermis.

The cerebellum is considered instrumental in correction of the motor error induced by inaccuracies in motor behavior. Such motor adaptation behavior was tested in animal models of dorsal vermis [52]. Takagi and colleagues trained non-human primates to perform saccades from 3.5° to the right to 6.5° to the left (i.e., 10°) [52]. At the time of onset of visually triggered saccades, the visual target disappeared and reappeared spontaneously at 3.5° to the left (i.e., 3° closer to the onset coordinates). The eyes however initially ended at programmed (6.5°) orientation causing 3° of movement error—subsequently, correction happened. These trials, when repeated several times, resulted in motor learning; the later trials (testing the adapted saccades) were smaller than initially planned 10° saccades. Such motor learning behavior was absent in immediate post lesion state, 1 week after ablation of dorsal vermis [52]. In addition, there was increase in the amplitude variability. Three months later, the animal regained adaptive capacity; however, the amplitude variability persisted [52].

The concept of structural motor reserve as noted in the animal models directly translates to acute onset of human disease such as in cerebellar stroke. In the acute phase, cerebellar stroke typically presents with nystagmus, saccade dysmetria, as well as hyperactive VOR and, on some occasions, skew deviation [63, 64]. The characteristics of nystagmus are variable, ranging from the gaze-evoked nystagmus to its combinations with various forms of horizontal or vertical spontaneous nystagmus, with or without its gravity-dependent modulation [65–67]. As expected from macaque lesion models, the ocular motor deficits in acute stroke patients should be mostly reversible. Unlike macaques that take months for resolution of ocular motor function, in typical instances the ocular motor deficits after acute stroke resolve in 48–72 h, despite sizable structural deficits noted under MRI [68–72]. How to explain such a disparity in time of functional reversal between macaque lesion experiments and human stroke patients? It is possible that after acute stroke there is incomplete loss of cerebellar tissue, and even the neural function that is lost is not permanent. Motor dysfunction in the hyperacute phase is likely due to a combination of cellular death as well as ischemic penumbra. The latter resolves with reperfusion over the course of hours, but damage from cellular death persists, hence appearing as rapid recovery from motor dysfunction. In contrast, in macaque experiments with total (or near total) excision, recovery takes longer in the absence of neural tissue.

**Fig. 2:**
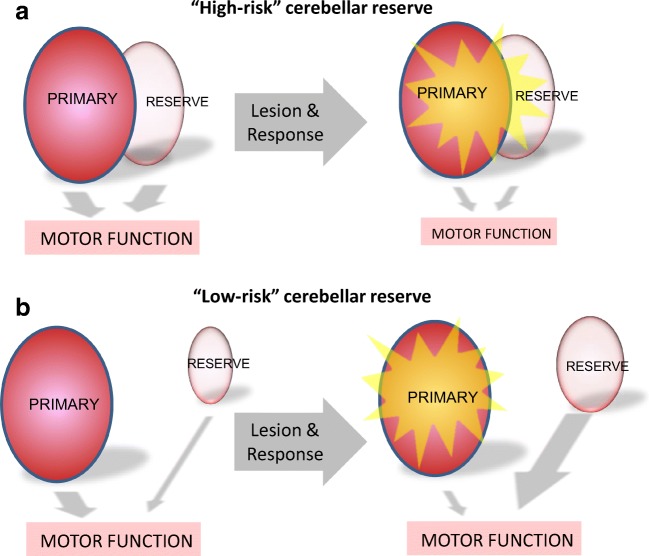
Two hypothetical types of cerebellar ocular motor reserve systems. **a** In high-risk cerebellar reserve, the neural substrate for the cerebellar reserve reside in proximity to the primary region. In such system, the lesion of primary substrate is also likely to affect parts of the reserve. As a result, there is poor reversibility of the lost motor function (or motor abnormality). In contrast, in low-risk cerebellar reserve, **b** the neural substrate for reserve is discretely located. In this system, the lesion to primary system does not affect the reserve, and there is an opportunity to compensate and reverse the motor function by upregulation of the reserve system

Patients with acute stroke and animal excision models allow us to speculate on two types of systems for structural ocular motor reserve. One type is where the “backup” neural tissue—the reserve—is anatomically adjacent to the primary substrate (Fig. 2a, e.g., dorsal vermis for pursuit eye movements or flocculus for the VOR). In this case, a sizable lesion may affect the primary site but would also lead to at least “partial” involvement of the “backup”—the reserve. In such cases, the chances for a sizable lesion to involve primary substrate and its reserve are both high—i.e., the “high-risk reserve”. In contrast the second type of system is where the “backup” substrate is anatomically discrete from the primary substrate (Fig. 2b). In such circumstances, even a sizable lesion of the “primary” location does not affect the brain’s capacity to recover using anatomically discretely located reserve—i.e., the “low-risk reserve.”

### Physiology of Adaptation and Maladaptation-Abnormal Consequences of Cerebellar Compensation

**Fig. 3:**
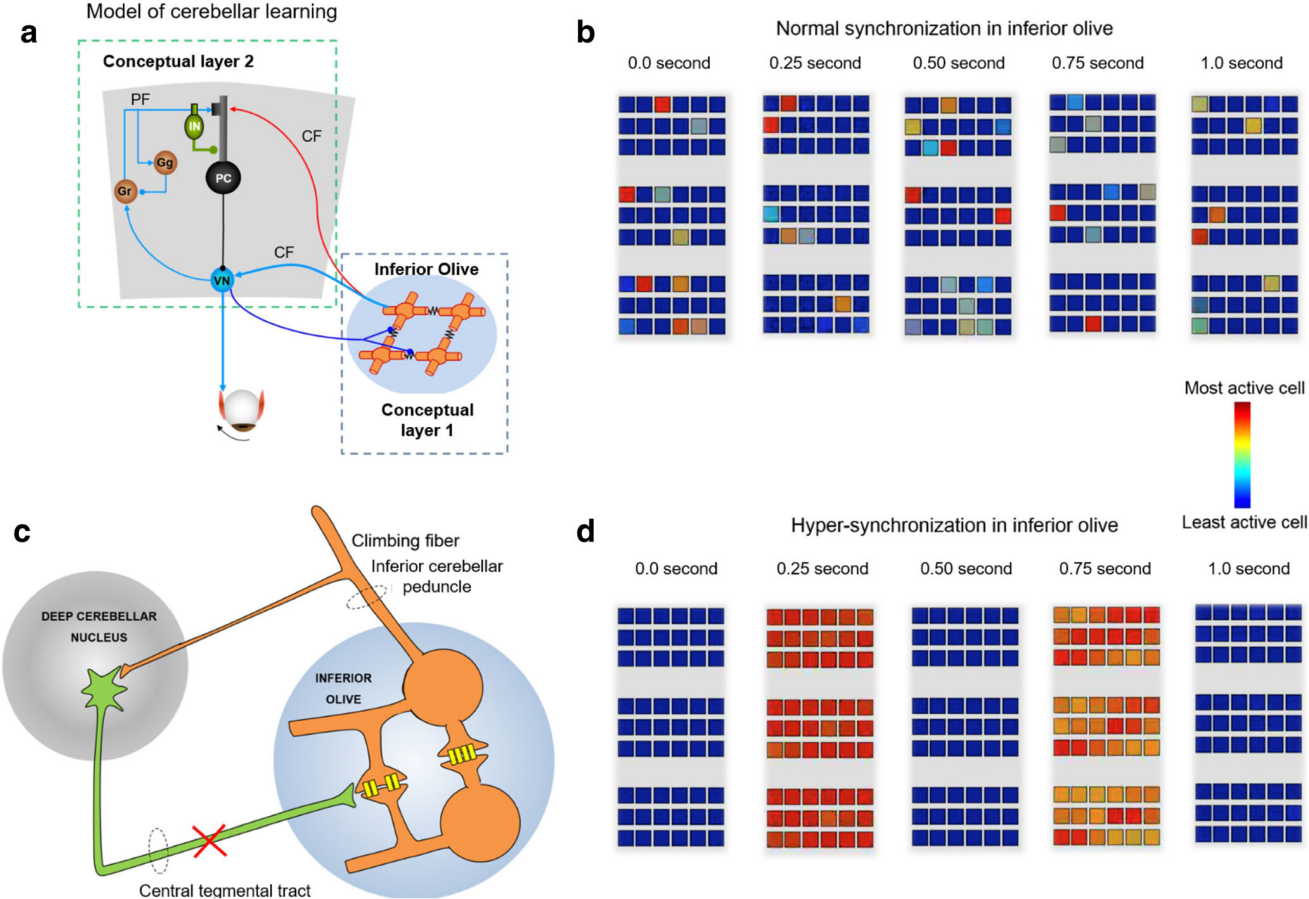
**a** A schematic representation of a model of classical delay conditioning. Inferior olive and cerebellar modules are illustrated. **b** Simulation of three groups of neuronal clusters of inferior olive in a healthy state, where the firing of the constituent neurons is out of synchrony. **c** Schematic depicting a breach in the continuity of the Guillain-Mollaret triangle that may lead to hypertrophy of inferior olive and a propensity for hypersynchrony. **d** Simulation of inferior olive discharge in the diseased state characterized by hypertrophic degeneration of inferior olive as seen in OPT. (Modified from Shaikh et al. Brain, 2010 [76]).

Structural cerebellar lesions, injury to the peripheral motor system or the changes in our environment increase the variability in the central neural processing (“noise”) for the motor command leading to dysmetria. Motor adaptation is one means by which cerebellum compensates for lost neuronal function. It then recalibrates the motor commands through error-based learning. Adaptation occurs via a neural circuit consisting of the inferior olive, cerebellar cortex, and deep cerebellar nuclei. Signals encoding errors in motor performance are relayed to the inferior olive and lead to a change in the phase of synchronous, sub-threshold oscillations in the membrane potential of neurons in this region [73, 74]. The discharge of the inferior olive carries this error signal to Purkinje cells in the cerebellar cortex via the climbing fibers and the deep cerebellar nuclei via climbing-fiber collaterals [75] (red and light blue arrows, Fig. 3a). Meanwhile, mossy fibers convey an internal copy of the performed motor command, via granule cells and parallel fibers, to the Purkinje cells [75, 77, 78] (light blue arrow, Fig. 3a). The convergence of activity of climbing fibers and parallel fibers leads to a change in the activity of Purkinje cells and deep cerebellar nuclei neurons, which appropriately signals future movements [75, 79–81].

In the healthy state, subsets of neurons in the inferior olive are coupled via gap junctions such that they exhibit synchronous subthreshold membrane-potential oscillations [82] and orchestrate synchronous complex-spike firing in the cerebellar cortex [83]. However, it is the incomplete neural coupling across the population that may be critical to convey information of sufficient quality to the cerebellum to be useful [84] (Fig. 3b). Excessive synchronization in the inferior olive can lead to rhythmic neural activity that might override any meaningful signals attempting to get through [85–87]. In their place, rhythmic but “meaningless” discharges from the inferior olive might trigger maladaptive learning in the cerebellum, causing abnormal oscillations of the eyes and other cranial nerve innervated structures as in the ocular palatal tremor syndrome (OPT) [76]—a degenerative syndrome that manifests after a breach in connections between the deep cerebellar nuclei and the inferior olive, typically the central tegmental tract (Fig. 3c). Such a disruption results in disinhibition and apoptotic hypertrophy of the inferior olive neurons (Fig. 3c), along with increased overall neural excitability due to the development of additional soma-somatic gap junctions between neighboring cells. We had shown in a mathematical model that the inferior olive under these conditions result in less random, more pulsatile and synchronous firing (Fig. 3d) that is transmitted downstream to the cerebellum to cause irregular, coarse oscillation of the eyes and palate in OPT. Aside from the eyes and palate oscillations, patients with OPT do not adapt to changes in motor commands as evidenced by the absence of saccade adaptation on long or short time-scale [85–87]. As a consequence, patients with OPT also present with ataxia despite no cerebellar structural deficits but due to abnormal (maladaptive) cerebellar learning process.

### Functional cerebellar ocular motor reserve

Acute deficiency of thiamine is known to cause a range of ocular motor deficits including decreased gain of the horizontal VOR, but relatively spared vertical VOR, gaze-evoked nystagmus, and mild ophthalmoparesis. It is believed that selective vulnerability of the periventricular grey neurons causes a range of ocular motor deficits in this classic manifestation of nutritional deficiency. Deficiency of thiamine results in glutamatergic neurotoxicity and subsequent cell damage, more vulnerably in the vestibular nuclei, causing the impairment in cerebellar function that is mediated by the brainstem vestibular nuclei. Fortunately, there is a therapeutic window over which supplementation of thiamine reverses the ocular motor dysfunction in patients with pre-encephalopathy stage of thiamine deficiency [88, 89]. The molecular and physiological underpinnings of thiamine deficiency and secondary functional reserve have been identified. The onset of neurological symptoms of thiamine deprivation (ataxia, loss of righting reflex) is accompanied by decreases in the activity of alpha-ketoglutarate dehydrogenase (alpha KGDH) in lateral vestibular nucleus and hypothalamus but to a lesser extent in medulla oblongata, striatum, and hippocampus, with no changes in other brain regions. Such decreases in alpha KGDH may explain decreased synthesis of glucose-derived neurotransmitters (acetylcholine, GABA, glutamate) in pyrithiamine-treated rat brain. Thiamine administration normalizes the defective alpha KGDH activity in all brain regions, restoring the neural dysfunction [90].

Immune-mediated cerebellar disorders are also examples of functional cerebellar ocular motor reserve. Suppression of hyperactive immune system or blockade of autoantibodies in early and acute phase of the immune mediated cerebellar syndrome typically results in successful treatment of immune mediated ocular motor deficits. Paraneoplastic disorders, in which cross-immunity to cancer cells and cerebellar or brainstem neuronal tissues cause ocular motor dysfunction such as nystagmus or ocular flutter, can have a dramatic presentation. These patients require immediate medical attention, and therefore these syndromes are usually diagnosed in their acute phase, not uncommonly before the diagnosis of the primary etiology is made (such as cancer). Rapid progression and prompt therapy frequently results in complete resolution of the ocular motor phenotype [91]. While in the acute phase, immune-mediated ocular motor dysfunction is nearly completely reversible. If it remains untreated, reversible cellular dysfunction due to auto-antibodies converts to irreversible clinical phenomenology. It is not infrequent in clinical practice to discover increased titers of anti-GAD antibody as a part of a diagnostic work-up of cerebellar degeneration or chronic unsteadiness and vertigo with simultaneous downbeat nystagmus. Such forms of chronic immune-mediated ocular motor dysfunction are typically challenging to treat. The mechanistic basis for conversion of reversible to irreversible immune-mediated cerebellar disorders are discussed in an earlier section. The same concept also applies to immune-mediated reversible (or irreversible) ocular motor reserve.

## Cerebellar Motor Reserve (Mitoma H, Kakei S, Manto M)

### Neural Mechanisms Underlying Cerebellar Motor Reserve: an Update of the Internal Model

#### Cerebellar Motor Reserve: Internal Models—Forward Models and Inverse Models

**Fig. 4:**
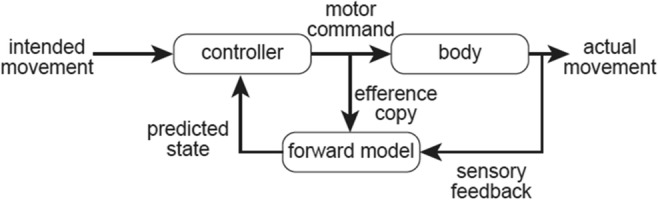
Schematic diagram of forward model

Feedback signals from the periphery have delays (~tens to hundreds of milliseconds) in reaching the brain. It is well known in engineering that feedback control based on the time-delayed inputs can result in unstable movements like ataxia. Ample evidence suggests the presence of neural mechanisms that overcome the delays in the cerebellum [92] to enable stable and dexterous control of movement. These neural mechanisms are collectively termed internal models, and are of two types; the forward model and inverse model [92] (Fig. 4). A forward internal model calculates a sensory prediction for an ongoing motor command, whereas an inverse internal model calculates a predicted motor command for a desired movement [92]. We recently demonstrated that activities of mossy fiber inputs to a hemispheric part of the cerebellum are transformed into outputs from the dentate nucleus which predict mossy fiber activities in the near future [93]. The internal model, with its predictive nature, is acquired through delicate motor learning processes [94]. Based on such learning processes, it is likely that any impairment of the cerebellar component(s) results in a new update or a reorganization of the management system operating the embedded internal model.

#### Neural Components for the Internal Model Update System: Synaptic Plasticity and Input Redundancy

The Marr-Albus-Ito theory is a representative hypothesis that explains the mechanisms responsible for acquisition of the internal model [94]. Ito assumed that learning signals, which are conveyed by the climbing fibers, modify parallel fiber-Purkinje cell synapses and also interneuron-Purkinje cell synapses so as to adjust the input-output organization of the cerebellum [94]. Thus, damage of the cerebellum induces an update of the internal model through the combination of synaptic plasticity. Another key neural mechanism involved in the update of the internal models is multiple convergence in a single microzone—a functional unit in the cerebellar cortex. A single mossy fiber, which conveys information from the periphery or the cerebral cortex, distributes axon terminals diversely in a medio-lateral direction, and innervates multiple longitudinal microzones [95]. Thus, various types of information, from both the periphery and the cerebral cortex, can be converged and integrated within a microzone. In other words, the highly redundant organization of mossy fiber inputs to cerebellar microzones must be beneficial for reorganization of the internal model within different microzones after impairment of one or more microzones responsible for the control. Taken together, any damage to the cerebellum triggers an update of the internal model using the combination of synaptic plasticity and redundant inputs. Thus, the internal model update must be an important process in recruitment of the cerebellar motor reserve.

### Therapeutic Strategies Based on Cerebellar Motor Reserve

#### Prospective Identification of Cerebellar Motor Reserve

Recovery from stroke or cerebellar trauma indicates the existence of self-recovery capacity within the cerebellum. Tailoring treatment to a particular patient, however, requires prospective identification of the extent of preservation of the cerebellar motor reserve. It is because preservation of the cerebellar motor reserve provides the basis for any recommendation regarding the introduction of neuromodulation therapy by the attending physician. A plausible technique for the assessment of the preserved cerebellar motor reserve may be analysis of the extent of cerebellar atrophy on MRI. It should be emphasized here, however, that preservation of specific cerebellar functions, such as ability for predictive control and motor learning, is a more reliable clinical index in the quantitative assessment of cerebellar motor reserve.

#### Ratio of Predictive Control to Feedback Control: an Index of Preservation of Cerebellar Motor Reserve

In pursuit of a smoothly moving target, one should use both predictive control and feedback control. Kakei et al. developed a method that quantitatively assesses preservation of predictive control in a smooth pursuit task [40]. In their experiment, the subjects were required to perform a smooth tracking movement of the wrist joint with a manipulandum, where the two degrees of freedom wrist position and surface EMG signals from four wrist prime movers were simultaneously recorded [96, 97]. The relationship between EMG signals and movement kinematics can be approximated with the following equation [96, 97].

$$ \tau (t)=\sum \limits_{i=1}^4{a}_i{T}_i(t)=M\ddot{\theta}+B\dot{\theta}(t)+ K\theta \left(\theta t\right) $$

where *τ*(*t*) denotes the wrist joint torque estimated in two ways (middle and right sides of the equation). *T*_*i*_(*t*) represents the tension of each muscle [96, 97] and *a*_i_ denotes parameters that convert the muscle tension into the wrist joint torque. The variables *θ*(*t*), $\dot{\theta}(t)$, and $\ddot{\theta (t)}$ represent angle, angular velocity, and angular acceleration of the wrist joint, respectively. *M*, *B*, and *K* represent the inertia [kg m^2^], viscous coefficient [N m s/rad], and elastic coefficient [N m/rad] of the wrist joint. We used a canonical correlation analysis (CCA) to determine the combination of *M*, *B*, *K*, and *a*_*i*_ (*i* = 1–4) that yielded the best regression for equation (1) (see detailed explanation in Lee et al. 2015 [96, 97]). It should be noted that with CCA*,* we cannot determine absolute values of these seven parameters. Instead, we can obtain only their ratios. Therefore, in the following paragraphs, we use *M*_*r*_, *B*_*r*_, and *K*_*r*_, instead of *M*, *B*, and *K* to emphasize that only their *ratios* are relevant [96, 97].

Our surprising observation was that the *B*_*r*_/*K*_*r*_ ratio can be used as an index of preservation of predictive control [40]. *B*_*r*_/*K*_*r*_ ratio represents how much velocity control is weighted relative to position control in muscle activities [96, 97]. For instance, in case of a simple feedback control to correct positional errors, velocity control is not necessary due to the lack of target velocity. In this case, *B*_*r*_/*K*_*r*_ ratio remains small. In contrast, in order to pursuit the moving target with a known velocity and position *in a predictive manner*, it is necessary to encode both the velocity and position of the target in the muscle activities, resulting in a much higher *B*_*r*_/*K*_*r*_ ratio. Thus, a decrease in the *B*_*r*_/*K*_*r*_ ratio (a decrease in velocity coefficient) suggests relative lack of predictive control in the tracking movement [40].

Using this rationale, we recently developed a way to quantify cerebellar motor preservation prospectively in patients with IMCAs and degenerative CAs [40].

1. The predictive control was preserved in IMCAs (i.e., relatively high *B*_*r*_/*K*_*r*_ ratio) but not in degenerative CAs (i.e., much lower *B*_*r*_/*K*_*r*_ ratio), although both groups of patients showed similar uncoordinated movement trajectories. In other words, patients with IMCAs performed the tracking task using predictive control, which was still preserved, but was inaccurate. On the other hand, for patients with degenerative CAs, predictive control was no longer available.
2. Notably, the conditions of these patients with IMCAs were considered in early stages, with no or mild cerebellar atrophy. Immunotherapy partially or completely improved the ataxias.

Overall, analysis of the *B*_*r*_/*K*_*r*_ ratio in ataxic patients may provide a unique and useful tool to find potentially treatable ataxias (i.e., ataxias with cerebellar motor reserve). However, to make this analysis more practical, it is necessary to develop improved methods for obtaining surface EMG recording from the four wrist prime movers. There is also a need to extend the analysis to movements of different joints, such as the elbow or knee, to evaluate the cerebellar motor reserve of different body parts.

Cerebellar neuronal circuitry is uniquely designed to generate spatiotemporally organized outputs. The cerebellar motor reserve is a mechanism to restore the organized outputs by reorganization of the cerebellar neuron circuitry, when it is damaged. Here, we demonstrated evidence of cerebellar motor reserve and reviewed its key elements. To make the most of the cerebellar motor reserve in patients with CA, it is desirable to start any treatment as early as possible when the cerebellar cell loss (i.e., cerebellar atrophy) is minimal or even undetectable. As a result, our challenge is to establish a reliable method for identifying a decrease in the functional cerebellar motor reserve physiologically rather than morphologically. This method can be applicable for quantification of structural cerebellar motor reserve.

In conclusion, any strategy for management of patients with CAs should be determined based on analysis and identification of cerebellar motor reserve before the administration of any treatment [10].

## Cerebellar Cognitive Reserve (Guell X, Schmahmann JD)

The cerebellum is a crucial node in the neural circuits that subserve cognition. Anatomical connections link the cerebellum to cerebral association and paralimbic regions and to other subcortical areas in thalamus and basal ganglia necessary for intellect and emotion [98–103]. Isolated cerebellar lesions are sufficient to generate deficits in executive function, language, visuospatial, social, and emotional processing (cerebellar cognitive affective syndrome, CCAS, also known as Schmahmann’s syndrome [104–107]). Neuroimaging experiments demonstrate cerebellar task activation and functional connectivity associated with cognitive control [108–118], and reveal cerebellar structural and functional abnormalities in neurological and psychiatric diseases that degrade thought and affect [119–123]. Here, we examine how this large and expanding body of literature informs our understanding of cerebellar cognitive reserve.

Behavioral and clinical investigations in humans reveal what may be regarded as cerebellar reserve in the domains of higher/nonmotor function. The original description of the CCAS noted improvements in verbal and visual memory, lexical access, visuospatial reasoning, visuomotor tracking, complex problem solving, and narrative writing when comparing patients suffering from an acute cerebellar lesion within weeks of the insult and 1–9 months after the insult [104]. Executive function continued to reveal the brunt of the neuropsychological impairment in follow up studies. Later investigations replicated the existence of cognitive improvement after cerebellar injury [124]. Studies examining the natural history of genetic cerebellar disorders indicate that cognitive deficits as measured by neuropsychological testing may be absent or minor in the early stages of cerebellar degeneration [125].

Neuropsychological testing experiments in patients suffering from injury or degeneration in the cerebellum have identified potential targets and strategies to enhance cognitive recovery in cerebellar dysfunction. A recent study evaluating 71 measures of cognitive function in 116 cerebellar patients identified that Trails Making, Go/No-go, Category Switching, Digit Span Backwards, Verb for Noun Generation, Work Stem Completion, and Phonemic and Semantic Fluency are among the most impaired neuropsychological measures in cerebellar dysfunction [107]. These cognitive domains might be optimal targets for exercises to improve cognitive function in cerebellar disease. Because patients with cerebellar injury often report difficulty multitasking [126], encouraging patients to focus on one rather than multiple thought operations at once may facilitate functional compensation. In line with this observation and the general understanding that the cerebellum modulates behavior around a homeostatic baseline, without conscious awareness, and according to context [99], recent reviews recommend the use of increased focus and conscious task awareness as a compensatory strategy in cerebellar injury [18, 127].

Neuroimaging and neuromodulation/neurostimulation experiments indicate that cerebellar compensatory reorganization might also be relevant in brain disorders not restricted to the cerebellum. Numerous studies have reported differences in cerebellar structure and function in a wide range of neurological and psychiatric disorders that degrade cognition and affect. Examples include Alzheimer’s disease [121, 128], frontotemporal dementia [121], Parkinson’s disease [123], autism spectrum disorder [119], schizophrenia [122], and major depressive disorder [120]. Some of these differences may not represent pathological changes in cerebellar structure or function, but rather compensatory reorganization changes that improve behavior. Behavioral differences associated with neuroimaging alterations can provide only correlational evidence to support this possibility. Compensatory reorganization changes are generally expected to correlate with improved behavioral function; although not necessarily, since it would also be reasonable to expect that compensatory mechanisms become more prominent as disease severity, and thus behavioral abnormalities, become more pronounced. Studies of neural stimulation might be able to provide causal evidence of cerebellar compensation in brain disorders, and indeed, recent studies in neuropsychiatry report behavioral improvement after cerebellar stimulation [129, 130]. Technological advancements that allow targeting small and deep brain structures [131–135] underscore the potential for cerebellar stimulation to become a useful therapeutic modality in the clinical treatment of cognitive dysfunction. Recent reports also suggest that it might be relevant to investigate surgical correction of gross cerebellar structural abnormalities such as Chiari malformation and posterior fossa arachnoid cysts as an additional cerebellar-focused intervention to relieve brain-wide dysfunction [136].

This large and expanding body of evidence describing cerebellar functional compensation in cerebellar-specific and brain-wide disorders highlights the need to address a fundamental question, namely, what mechanisms subserve cerebellar cognitive reserve? Anatomical and neuroimaging investigations that characterize the normal structure and function of cerebellar and cerebello-cerebral circuits provide a basic scientific framework to address this question, as follows.

1. Cytoarchitecture is essentially uniform throughout the cerebellar cortex. There might thus be a uniform computation subserving all cerebellar functions (universal cerebellar transform theory, UCT) [99, 137–140]. Consequently, a similar mechanism might underlie both motor and cognitive cerebellar reserve. Numerous questions remain open regarding the characterization and potential enhancement of cerebellar cognitive reserve at all levels of molecular, cellular, and systems neuroscience. The UCT theory might contribute to these investigations by guiding future research towards the description of mechanisms and development of interventions that are similar across motor and non-motor domains.
2. Specific cerebellar territories are anatomically linked to specific extracerebellar regions [98–103]. Functional connections as indexed by fMRI in humans validate this observation [108, 109, 111, 116–118]. These anatomical and functional relationships between cerebellum and the remainder of the neuraxis allow the UCT to access distinct streams of motor and cognitive information processing, enabling cerebellar compensation of extracerebellar function in neuropsychiatric diseases that may not primarily affect the cerebellum. Extracerebellar functional networks such as default-mode network are anatomically and functionally coupled to cerebellar territories that are specialized in similar processes. In this way, cerebral cortical alterations in specific functional networks may be matched by compensatory cerebellar changes in the same functional networks (e.g., see Guo et al. [121, 141] showing network-specificity in cerebral cortical and cerebellar changes in Alzheimer’s disease and frontotemporal dementia).
3. Feedforward cerebro-cerebellar connections link associative and paralimbic regions to cerebellum in a manner closely matching the feedback cerebello-cerebral circuits, perhaps forming closed loops [102], with some exceptions [142]. These reciprocal connections allow the cerebral hemispheres to influence cerebellar function, making it reasonable to consider that extracerebellar compensation exists in cases of cerebellar injury. The cerebellum modulates behavior *around a homeostatic baseline*, *without conscious awareness*, *and according to context* [99]*.* Compensatory cognitive strategies based on increased focus and awareness [18, 127] resonate with the possibility of top–down cognitive processing in cases of cerebellar dysfunction, drawing on cerebral hemispheric direction of cognitive domains deprived of cerebellar automaticity. Lesion studies predating modern cerebellar anatomical and functional neuroscience presaged this possibility, as cerebral cortical ablation in monkeys with concomitant cerebellar lesions produced a decompensation of initially recovered behavior [21].
4. Cerebello-cerebral connections define functional territories within the cerebellar cortex. There are two regions of motor representation located in lobules I–VI and VIII [143–145], and three regions of cognitive representation located in lobules VI/Crus I, Crus II/VIIB, and IX/X [108, 109]. Multiple specific cognitive domains such as working memory and default-mode processing are independently mapped within each of these three territories of cognitive representation. The knowledge that the cerebellum contributes to cognition in addition to motor control, together with the realization that most of the cerebellar cortex is devoted to the control of cognitive rather than motor functions, resolves the old misconception that large cerebellar lesions often result in no neurological symptoms. Once thought to represent a paradigmatic example of cerebellar reserve, we now know that cases of large cerebellar lesions with no motor deficits correspond in fact to lesions of cognitive-specific cerebellar territories, i.e., posterior cerebellum irrigated by the posterior inferior cerebellar artery, that produce cognitive impairment, but no motor symptoms [146, 147].
5. The restoration of intrinsic cerebellar histological specificity and input-output organization is vital to cerebellar reserve [48, 148]. The central tenet of the dysmetria of thought theory is the duality of the universal cerebellar transform subserved by the constant architecture of the cerebellar cortex, set against the heterogeneity of cerebellar connections with cerebral hemisphere and other extracerebellar structures [99, 137–140]. In addition to the macroscale systems approach to cerebellar reserve, therefore, the cell types and organization of cerebellar histology are critical to the concept of cerebellar reserve for both cognition and movement. Different types of neurons and glia are affected differentially in terms of order and rate of involvement in the spinocerebellar and other neurodegenerative ataxias [149]. Information is available regarding the role of granule cells in reward, for example [150], and the role of mossy fibers in conveying context and of climbing fibers for detection of errors and for learning [94, 151]. But in the context of attempted neural circuit reorganization in the setting of the programmed cell death that defines neurodegenerative ataxias, it is likely that Purkinje neurons, granule cells, interneurons, nuclei, and afferent and efferent pathways play different roles in the clinical manifestations. Details of the temporal order, nature, and complexity of neuronal pathology in the neurodegenerative ataxias are surely essential to the impairments in cognition and emotion, or the lack thereof early in the course when functional circuit integrity may be maintained through neuronal and architectural plasticity. Future study may shed light on these critical details. Acute macroscopically identifiable injuries such as ischemic or hemorrhagic strokes, space-occupying lesions, and the cerebellar atrophy of immune ataxias exemplify less targeted pathology. Here, the cerebellar reserve for cognition may be determined both by the combination of potential neural microcircuit reorganization within cerebellum itself, as well as the details of the mesoscale cerebellar connections with the remainder of the neuraxis.

Taken together, contemporary cerebellar systems neuroscience resolves earlier misconceptions and attributes new meanings to the concept of cerebellar reserve, supports the existence of cerebellar cognitive resilience and recovery in cerebellar and cerebellar-linked disorders, hints at potential targets and strategies to enhance cerebellar cognitive recovery, and provides a basic scientific framework to study the mechanisms underlying these observations. Future work in this field will contribute to the expanding appreciation of the cerebellum as central to the evolving science and medicine of human cognition.

## Cellular Mechanisms Underlying Functional Cerebellar Reserve: From Autophagy to Motor Training (Fucà E, Buffo A)

Autophagy is a conserved catabolic process that delivers the cytosol and cytosolic constituents to the lysosome. It has a fundamental role in the maintenance of cellular homeostasis and in the protection of cells from various insults, including misfolded proteins and damaged organelles [152]. Neurons may be particularly dependent on efficient autophagy to sustain their integrity and functions, due to the need to constantly monitor the quality of proteins and mitochondria along axons, which is critical for proper neuronal function, and to sustain high-energy demands and protein turnover at the synapses. Neurons may also take advantage of autophagy for synaptic plasticity and memory enhancement [153]. Yet, different neuronal types appear to display different degrees of dependency on autophagy. Notably, abrogation of major autophagic flux in animal models revealed a specific sensitivity of Purkinje cells, which underwent fast degeneration compared to other neurons [154]. However, consistent with its homeostatic nature, also autophagy hyperactivation induced neurotoxicity in Purkinje neurons [155]. Thus, both excessive or insufficient autophagic flux can promote neural cell dysfunctions and death [156, 157]. It is thus plausible that, depending on the pathological insult, increased or decreased autophagy supports protective effects, and possibly sustains plastic changes and compensatory responses in circuits, thereby incrementing brain reserve.

Significant dysfunctions in the autophagy pathway have been identified in several neurodegenerative diseases [158, 159]. Importantly, studies on both patients [160–162] and preclinical experimental models [160, 161, 163–165] indicated that alterations of the autophagic flux are implicated in different types of cerebellar ataxia. In particular, some forms of spinocerebellar ataxias (SCA1-3; 6,7,17) and dentatorubral-pallidoluysian atrophy are caused by the abnormal CAG repeat expansion [166], resulting in extended polyglutamine (polyQ) tracts, aggregates, and intracellular inclusions, which are a substrate of autophagy-mediated degradation. However, in these pathologies, autophagy fails to remove toxic molecules due to their abundance and to the inhibitory effects of long polyQ mutation on mechanism initiating autophagy [167]. Consistently, pharmacological treatments targeting autophagy provided beneficial effects in several models of hereditary cerebellar ataxia [168–171]. Moreover, positive outcomes associated with changes in autophagy were also induced by motor training administered at presymptomatic stages in the *tambaleante* (tbl) mouse model of ataxia [172]. In tbl mice [173], alteration of the HERC1 E3 ubiquitin ligase leads to overactive autophagic processes resulting in massive degeneration of Purkinje neurons [163]. Motor exercise favored tbl Purkinje neuron survival, reduced atrophy, increased afferent contacts to mutant Purkinje neurons, and allowed partial circuit stabilization, with moderate positive motor consequences [172]. This was accompanied by autophagy attenuation and increased BDNF, a key signal underlying both neuroprotection and neural plasticity [174]. These data suggest that, through autophagy modulation, early motor rehabilitation at the stage of functional disorder (for instance in cases when familiar transmission allows an early genetic diagnosis) can delay the manifestation and progression of the pathology. Beneficial effects can result from facilitating cerebellar reserve both in terms of activation of neuroprotective mechanisms, and of stimulation of circuit maturation. It is worth noting that the impact of motor exercise on autophagy regulation with neuroprotective effects has been shown also in other neurological pathologies [175–177]. Autophagy modulation can therefore add to the numerous mechanistic substrates through which experience—including exercise—molds the brain. Motor exercise is in fact proposed to stimulate the improvement of symptoms through cellular and molecular modifications contributing to the enrichment of neural reserve including synaptogenesis, neurogenesis, gliogenesis, and angiogenesis [178]. Such cellular changes are sustained by molecular modifications involving neurotransmitters and neurotrophins, likely supporting neuroprotection. Indeed, motor training can stimulate neuroprotective mechanisms [179] and alleviate ataxic symptoms in both preclinical and clinical studies also at stages of overt ataxic pathology [180–183] and, in general terms, in other neurodegenerative disorders [184, 185]. However, in cerebellar pathologies, the extent of exercise-dependent amelioration, as well as its endurance, is affected by factors such as disease severity and areas of neurodegeneration, and becomes more limited with the progression of the pathology [186–188]. Taken together, these observations enforce the use of motor exercise in ataxia to implement cerebellar reserve and promote a delay in the progression of the disease.

## Potentiation of Cerebellar Reserve Through Complex Environmental Experiences: Modifications of Synaptogenesis (Petrosini L, Gelfo F)

The notable neuroplastic properties of cerebellar circuits allow them to encode experience and learn behaviors in physiological conditions, and even to compensate for deficits induced by a lesion. One of the main cerebellar plastic mechanisms works by modifying density and size of Purkinje cell dendritic spines (Pcds), which receive the entire afferent information that arrives to the cerebellar cortex and then are adaptively controlled by environmental factors [189–191]. Spine changes in turn regulate synaptic strength and signaling properties.

### Lesion-Induced Modifications of Synaptogenesis

**Fig. 5:**
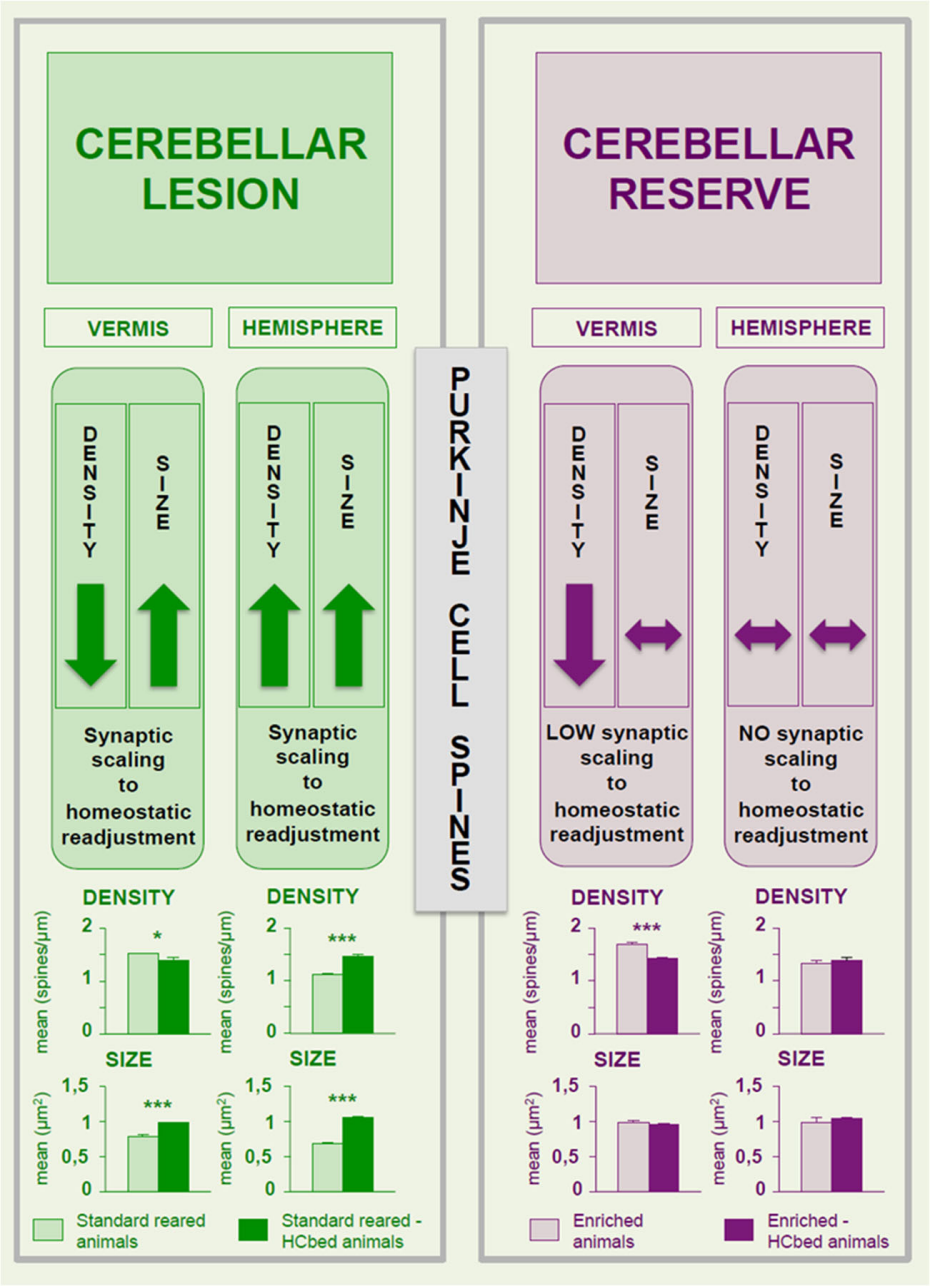
Lesion- and environmental enrichment-induced modifications of synaptogenesis. Effects of hemicerebellectomy (HCb) in standard reared animals (on the left) and in enriched animals (on the right) on Purkinje cell dendritic spines. The changes occurring in density and size of Purkinje cell spines of vermis and hemisphere are reported. Upper histograms show mean spine density in Purkinje cell distal dendrites. Lower histograms show mean spine area in Purkinje cell distal dendrites. Asterisks indicate significant differences (Tukey’s post hoc comparisons [20]) between groups: *p < 0.05; ***p < 0.0005. Data are shown as mean ± S.E.M. *HCbed* hemicerebellectomized

Following unilateral cerebellar ablation (as in the case of HCb), the lesioned animals reared in standard conditions succeeded in almost completely compensating for postural and locomotor symptoms, maintaining only slight ataxic symptoms, and conversely never fully recovering from complex behavioral and spatial deficits [192]. This behavioral pattern is accompanied by Pcds density decreased in the spared hemivermis and increased in the spared hemisphere [20]. Furthermore, in both regions, spine area (surface of the spine from the dendritic surface to the spine ending) and head diameter (diameter of the spine bulbous ending) are enhanced (Fig. 5). In the spared hemivermis, the lesion-induced spine response appears to be a compensatory reaction, aimed at readjusting the baseline firing rate set-point [193–195]. Namely, the partially deafferented vermian Purkinje cells may compensate for the reduced parallel fiber excitatory drive by sensing global levels of activity and operating a homeostatic synaptic scaling (resulting in diminished spine density), given the diminished input cannot sustain a large number of excitatory connections [193–195]. The decreased spine number results in upregulated excitatory signaling and enhanced synaptic efficacies (resulting in enhanced spine size and then synapse strength). Even in the spared hemisphere, the spine response (enlarged spines in a pattern of enhanced density) is an advantageous response elicited by the increased activity in the neuronal projections bidirectionally connecting cortical and subcortical regions with the spared cerebellar hemispherical regions, once again to maintain the homeostatic condition.

### Lesion-Induced Modifications of Synaptogenesis in the Presence of Complex Environmental Experiences

Are the lesion-induced morphological plastic changes modulated by the presence of an enhanced cerebellar reserve? To increase the reserve to be spent in the presence of an insult, animals may be exposed to environmental enrichment (EE) before the cerebellar lesion. In fact, environmental conditions that enhance sensory, motor, cognitive, and social stimulations influence structure and function of brain in general, and of cerebellum in particular [178, 196, 197]. Specifically, in healthy animals, EE induces improvement of motor and spatial performances [198] and increase in Pcds density and size [199]. In hemicerebellectomized (HCbed) animals, a prolonged pre-lesional EE accelerates the compensation of postural and locomotor deficits elicited by the HCb, and makes the spatial performances of lesioned animals similar to those of intact animals. This behavioral pattern is accompanied by the maintenance of almost all features in Pcds density and size induced by EE, without most of the plastic rearrangements induced by the lesion [20, 200–202], with the only exception for the reduced Pcds density in the hemivermis, a region minimally responsive to EE influence [199]. Specifically, in comparison with healthy enriched animals, the enriched HCbed animals show decreased spine density in the hemivermis and maintenance of the EE-induced increased spine density in the hemisphere, while in both cerebellar regions the EE-induced increase in spine area and head diameter is maintained (Fig. 5). These findings provide support for the conclusion that the optimal shaping of neuronal connectivity is reached in a cerebellum that has been environmentally enriched. The presence of pre-lesional shaping of neuronal morphology may limit the occurrence of post-lesional re-adjustment, possibly because further re-arrangement in response to damage is not needed because the neural networks are already optimized.

The large spines induced by EE abundantly express AMPA receptors and are associated with large postsynaptic density, producing consequently large AMPA-mediated currents [203–207]. Furthermore, the large spines are persistent and functionally stronger in their response to glutamate and regulation of intracellular calcium, and show an increased LTP [189, 205]. Consistently, the large enriched Pcds are more stable and less susceptible to homeostatic re-adjustments. The maintenance of the EE-induced neuronal strengthening allows enriched HCbed animals to better respond to the lesion and supports their accelerated functional recovery. Molecular factors promoting such neuronal strengthening include the neurotrophins, which potentiate neuronal activity and synaptic connections [208]. However, in enriched HCbed animals, EE exposure does not influence BDNF levels in the spared hemicerebellum, but upregulates BDNF expression in frontal cortex [209]. Accordingly, EE induces increased spine density in neocortical frontal and parietal pyramidal neurons [210]. Furthermore, in enriched HCbed rats, EE promotes the efficiency of the cortico-subcortical circuitry by counteracting the lesion-induced shrinkage of fast-spiking striatal interneurons [211, 212]. In conclusion, the re-arranged and tuned cerebellar circuits that are the basis of cerebellar reserve appear to work in synergy with the re-arranged circuits of the whole brain. Greater functional connectivity is therefore likely to enable more successful adaptive responses.

## Conclusion

Cerebellar reserve, defined as the capacity for compensation and restoration in response to disease, is a treatment-based framework. By introducing this framework, we hope to facilitate the rational planning of treatment strategies.

Cerebellar reserve may be conceptualized as the result of two complementary mechanisms. When the underlying etiology elicits acute and focal structural damage, the degraded cerebellar function may be compensated for by other cerebellar or by extracerebellar areas (structural cerebellar reserve). When the pathological changes compromise cerebellar neuronal integrity gradually leading to cell death, it is possible that the affected area itself can compensate for the slowly-evolving cerebellar lesion (functional cerebellar reserve).

### Prediction of Prognosis After Cerebellar Insults

Prognosis of cerebellar lesions/diseases can be predicted based on the preservation of cerebellar reserve. In cerebellar ocular reserve, prognosis is correlated with the type of impaired ocular movements. Accumulated clinical observations on cerebellar ocular deficits can predict prognosis, from good adaptation to maladaptation. Conversely, in cerebellar motor reserve, the extent of preservation of cerebellar reserve can be quantified by using an index of cerebellar predictive controls, the ratio of B/K obtained from the equation of motion in tracking tasks. Cerebellar cognitive reserve reflects the temporal features of the lesion—age of patient at the time of disease onset and temporal course of the disease, and neuroanatomical details of the lesion—both at the level of the microcircuit, and the mesoscopic details of cortico-nuclear involvement and disruption of cerebrocerebellar afferent and efferent linkage.

### Therapeutic Strategies in Cerebellar Disorders

#### Curative Treatment

**Fig. 6:**
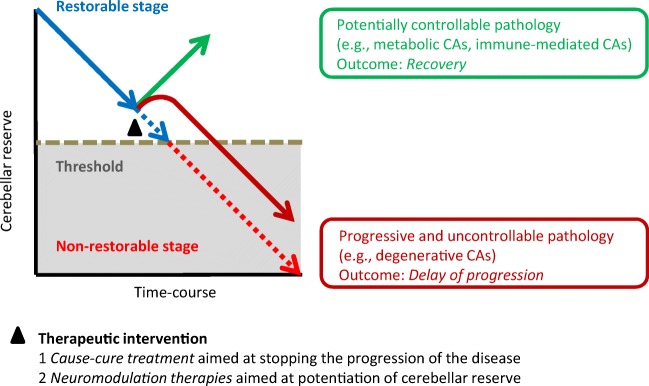
Schematic diagram of therapeutic strategies. Early therapeutic intervention at the time when cerebellar reserve is preserved is highly recommended. The therapeutic options include *Curative treatment*, which aims to stop disease progression, and *Neuromodulation therapies* designed to potentiate the cerebellar reserve. Treatment outcome will vary according to the pathology. When pathologies are potentially controllable (e.g., metabolic and immune-mediated CAs), CAs will improve. When pathologies are progressive and uncontrollable, these therapies can delay the disease progression. *CAs* cerebellar ataxias

The aim of curative therapy is to stop/minimize continuation of the pathological process (Fig. 6). For those cerebellar disorders that can be treated emergently, therapy must be introduced while cerebellar reserve is still present. Clinicians are urged to not miss this window of opportunity. We have stressed the importance of early diagnosis and treatment by advancing the term “Time is Cerebellum” [213, 214], based on the phrase “Time is Brain”, a motto used to stress the importance of early intervention in ischemic brain diseases [215].

#### Potentiation of Cerebellar Reserve

The potentiation of cerebellar reserve is the second option for treatment (Fig. 6). A powerful tool to enhance cerebellar reserve is the exposure to complex environmental stimulations, as occurring in the EE paradigm. Among the multiple cognitive functions beneficially affected by enhanced environmental stimulations, cognitive flexibility stands out as a domain that is heavily reliant on cerebellar circuitries [101, 104, 109] and which enables the individual to effectively change behavior in response to the needs of the environment, even in the presence of brain pathologies [216]. It is no coincidence that this flexibility exploits two cerebellar neuronal properties: multifaceted synaptic plasticity, and redundant information processing [10]. By modulating these cerebellar features, motor and cognitive rehabilitation as well as noninvasive cerebellar stimulation may represent effective therapeutic options enhancing cerebellar flexibility [10]. In addition, we anticipate that neurotransplantation might become an option for potentiation of cerebellar reserve, by prolonging neuronal function.

#### Beyond Cerebellar Disorders: Compensation and Restoration in Brain-Wide Disorders

Anatomical and functional connections link the cerebellum to cerebral cortical association and paralimbic regions and to subcortical areas in the thalamus and the basal ganglia. Consistently, a wide range of neurological and psychiatric disorders are associated with structural or functional differences in the cerebellum (some of which might represent compensatory rather than pathological changes), and behavioral impairments are improved after cerebellar stimulation in some neuropsychiatric disorders. Thus, cerebellar compensatory reorganization might also be relevant in brain disorders not restricted to the cerebellum.

By introducing the concept of cerebellar reserve, we hope to advance the rational approach to therapeutic strategies in patients with cerebellar and brain-wide disorders.
